# Synergistic antitumor activity of regorafenib and rosuvastatin in colorectal cancer

**DOI:** 10.3389/fphar.2023.1136114

**Published:** 2023-04-17

**Authors:** Tao Yuan, Ruilin Wu, Weihua Wang, Yue Liu, Wencheng Kong, Bo Yang, Qiaojun He, Hong Zhu

**Affiliations:** ^1^ Institute of Pharmacology and Toxicology, Zhejiang Province Key Laboratory of Anti-Cancer Drug Research, College of Pharmaceutical Sciences, Zhejiang University, Hangzhou, China; ^2^ Department of Gastroenterological Surgery, Affiliated Hangzhou First People’s Hospital, Zhejiang University School of Medicine, Hangzhou, China; ^3^ Innovation Institute for Artificial Intelligence in Medicine, Zhejiang University, Hangzhou, China; ^4^ Cancer Center of Zhejiang University, Hangzhou, China; ^5^ Second Affiliated Hospital, Zhejiang University School of Medicine, Hangzhou, China

**Keywords:** regorafenib, rosuvastatin, statins, MAPK signaling, colorectal cancer

## Abstract

**Introduction:** Colorectal cancer is one of the most prevalent life-threatening malignant tumors with high incidence and mortality. However, the efficacy of current therapeutic regimens is very limited. Regorafenib has been approved for second- or third-line treatment of patients who are refractory to standard chemotherapy diagnosed with metastatic colorectal cancer, but its clinical efficacy needs to be further improved. Accumulating evidence demonstrates that statins also possess potent anticancer activities. However, whether regorafenib and statins pose synergistic anticancer effects in colorectal cancer is still unclear.

**Methods:** Sulforhodamine B (SRB) assays were applied to evaluate the anti-proliferative activity of regorafenib or/and rosuvastatin *in vitro*, and immunoblotting analysis were applied to detect the effects of regorafenib/rosuvastatin combined treatment on mitogen-activated protein kinase (MAPK) signaling and apoptosis-related proteins. MC38 tumors were applied to investigate the synergistic anticancer effects of regorafenib in combination with rosuvastatin *in vivo*.

**Results:** We found that regorafenib in combination with rosuvastatin exerted significant synergistic inhibition against colorectal cancer growth *in vitro* and *in vivo*. Mechanistically, regorafenib and rosuvastatin combination synergistically suppressed MAPK signaling, a crucial signaling pathway promoting cell survival, as indicated by the reduction of phosphorylated MEK/ERK. In addition, regorafenib in combination with rosuvastatin synergistically induced the apoptosis of colorectal cancer *in vitro* and *in vivo*.

**Discussion:** Our study demonstrated the synergistic anti-proliferative and pro-apoptotic effects of regorafenib/rosuvastatin combined treatment in colorectal cancer *in vitro/vivo* and might potentially be evaluated as a novel combination regimen for clinical treatment of colorectal cancer.

## 1 Introduction

Colorectal cancer (CRC) has emerged as the third most common cancer and the second leading cause of cancer-related deaths throughout the population worldwide (Sung et al., 2021). Currently, surgical resection is the primary resort modality for localized early-stage colorectal cancer, while advanced colorectal cancer mainly adopts a strategy of combining chemotherapy with targeted drugs (Dekker et al., 2019; Xie et al., 2020; Biller and Schrag, 2021). Combination therapy with chemotherapeutic and targeted drugs provides notable survival benefits in clinical practice, which to some extent overcomes the disadvantages of former such as poor efficacy, long-term course of treatment and great undesirable side effects, together with the vulnerability of the latter like off-target possibility (McQuade et al., 2017). Nevertheless, the 5-year survival rate of patients with advanced colorectal cancer is still only 12%, indicating the urgency of developing more effective intervention strategies (Siegel et al., 2019).

Regorafenib (BAY 73-4506, Stivarga^®^), a novel diphenylurea multi-kinase inhibitor, is capable of blocking activities of multiple protein kinases, including VEGFR1-3, TIE2, PDGFR-β, FGFR, RAF, *etc.* (Grothey et al., 2020). The dual blockade of VEGFR and TIE2 endows regorafenib with the simultaneous anti-angiogenesis and vascular normalization effects. Additionally, regorafenib curbs molecular pathways of escaping VEGF inhibition by targeting PDGRF and FGFR, exerting a sustained anti-angiogenic effect. More researches delving deeper into antitumor mechanisms of regorafenib revealed that the broad-spectrum of kinase inhibition allows regorafenib to regulate a wide range of cellular processes including proliferation, apoptosis, and migration of cancer cells, as well as immunosuppression and remodeling of tumor microenvironment (Arai et al., 2019; Wu et al., 2019). As the first small molecular multi-kinase inhibitor gaining survival benefits in metastatic colorectal cancer (mCRC) progressed after all standard therapies, regorafenib was approved for treating colorectal cancer by Food and Drug Administration (FDA) in 2012 (Demetri et al., 2013; Grothey et al., 2013). Although regorafenib displayed a degree of clinically therapeutic potential, its efficacy yet remained to be improved (Sanoff et al., 2018; Xue et al., 2018). Therefore, development of novel combinational therapies to enhance the efficacy of regorafenib and overcome its resistance is critical to broaden its targeted usage.

Statins are the inhibitors of 3-hydroxy-3-methylglutaryl coenzyme A reductase (HMG-CoA), an enzyme accountable for the rate-limiting step of cholesterol synthesis (Kazi et al., 2017). Statins competitively inhibit HMG-CoA reductase to diminish mevalonate production, which leads to an increase in low-density lipoprotein (LDL) receptors and then gives a boost of LDL catabolism (Istvan and Deisenhofer, 2001). Consequently, the endogenous synthesis of cholesterol is interfered. Additionally, statins are known as more comprehensive lipid-lowering agents because of the ability to lessen triglycerides and raise high-density lipoprotein (HDL) levels to some extent (Liu et al., 2019a; Korani et al., 2019). Over recent years, the antitumor activities of statins have been discovered gradually. Massive clinical and epidemiological studies have reported the antitumor properties of statins: a pooled analysis of two clinical trials concluded that the oncology patients using statins displayed better overall survival (OS) and progression-free survival (PFS), among metastatic pancreatic cancer patients who had received first-line chemotherapy (Abdel-Rahman, 2019); Li *et al.* noticed a strong relationship between statin usage and a significant reduction of overall mortality and lower cancer specific mortality in CRC (Li et al., 2021).

Several studies have revealed the antitumor mechanisms of statins and demonstrated that statins modulate a series of cellular activities, including autophagy, proliferation, metastasis and apoptosis, and shape tumor microenvironment (Yang et al., 2017; Mehibel et al., 2018), well suggesting the therapeutic potential of statins in CRC. Furthermore, statins appear to maximize the efficacy and effectively overcome the defects of conventional cancer therapies. Recently, McGregor *et al.* demonstrated that simvastatin-mediated oxidative stress generation enhanced cellular apoptosis induced by mitogen-activated protein kinase (MEK) inhibitors in pancreatic cancer cells (McGregor et al., 2020). Iannelli *et al.* identified the synergistic antitumor effect of valproic acid/simvastatin combination in tackling the progression and therapeutic resistance of metastatic castration-resistant prostate cancer (mCRPC) via mevalonate pathway/YAP axis (Iannelli et al., 2020). Collectively, these studies demonstrated the crucial roles of statins in combined therapies and suggested the novel antitumor combination regimen needed further exploring.

In the present study, we demonstrated that regorafenib in combination with rosuvastatin synergistically inhibited colorectal cancer growth by suppressing mitogen-activated protein kinase (MAPK) signaling and promoting the apoptosis of colorectal cancer *in vitro* and *in vivo*. Our study demonstrated the synergistic anti-proliferative and pro-apoptotic effects of regorafenib/rosuvastatin combined treatment in colorectal cancer and provided a novel combination regimen for clinical treatment of colorectal cancer.

## 2 Materials and methods

### 2.1 Cell lines and cell culture

Cell lines SW620 and MC38 were purchased from the Cell Bank of the Chinese Academy of Sciences (Shanghai, China). Both SW620 and MC38 cells were cultured in RPMI-1640 medium supplemented with 10% fetal bovine serum (FBS), penicillin (100 U/mL) and streptomycin (100 U/mL). All cells were maintained at 37°C in a humidified atmosphere containing 5% CO_2_.

### 2.2 Reagents and antibodies

Regorafenib (HY-10331), Rosuvastatin Calcium (HY-17504) and Z-VAD-FMK (HY-16658B) were purchased from MedChemExpress. Both regorafenib and rosuvastatin were dissolved in sterile dimethyl sulfoxide (DMSO). Stock solutions (50 mM) were kept at −80°C and diluted in culture medium to working concentration before experiment. The antibodies against phospho-MEK (#9154), MEK (4694S), phospho-p44/42 MAPK (#4370) and p44/42 MAPK (#4695) were purchased from Cell Signaling Technology. The antibodies against cleaved PARP (ET1608-10) were purchased from HuaBio Bioscience, the antibodies against GAPDH (db 106) were purchased from Diagbio Bioscience.

### 2.3 Immunoblotting analysis

CRC cells were seeded into 12-well plates at a density of 10^6^ cells/well. For cellular apoptosis experiment, adherent cells were incubated with indicated drugs for 24 h, while for signaling pathway detection, cells were exposed to drugs for 4 h and subsequently stimulated with 500 ng/mL EGF for 30 min or not. Then, cells were harvested and lysed in 2.5× loading buffer. Lysates were boiled at 95°C for 25 min and size-fractionated using SDS-PAGE and then transferred to polyvinylidene fluoride (PVDF) membrane (Millipore, Bedford, UK). Having blocked with 5% milk dissolved in phosphate-buffered saline with Tween 20 (PBS-T) for 1 h, membranes were incubated with primary antibodies: PBS-T (v/v, 1:1000) at 4°C overnight. Then membranes were washed by PBS-T for three times (10 min/time), followed by incubation with secondary antibodies: PBS-T (v/v, 1:5000) at room temperature for 1 h. Then membranes were washed by PBS-T for three times (10 min/time) again. Finally, the membranes were incubated using enhanced chemiluminescence (ECL) substrate (PerkinElmer, Inc., Waltham, United States of America), exposed and visualized on Amersham^TM^ ImageQuant 800.

### 2.4 Anti-proliferative activity *in vitro*

SW620 and MC38 cells were seeded 2000 cells per well in 96-well dishes. After static adherence, cells were exposed to different concentrations of regorafenib and/or rosuvastatin for certain times, followed by Sulforhodamine B (SRB) assays. Inhibition ratio (IR) of SRB: IR _SRB_ = 100% - (OD value _treatment group_-OD value _blank_)/(OD value _control group_-OD value _blank_) × 100%. The coefficient of drug interaction (CDI) analysis was conducted to quantitatively evaluate drug interaction, as synergism (CDI <1), additivity (CDI = 1) and antagonism (CDI >1), and especially, CDI <0.7 represents a significantly synergistic effect (Damon and Cadman, 1986; Cao and Zhen, 1989; Sagwal et al., 2018; Luchtel et al., 2020). CDI = XY/(X×Y), where XY is the ratio of the combination group to the control group and X or Y is the ratio of the single drug group to the control group, based on the absorbance of each group.

### 2.5 Antitumor activity *in vivo*

Female C57BL/6 mice were purchased from National Rodent Laboratory Animal Resource (Shanghai, China). All animal experiments were conducted in accordance with the regulations of Institutional Animal Care and Use Committee (IACUC). The protocols for the animal study were approved by the Animal Care and Use committee of Zhejiang University, with the ethical approval numbers IACUC-s22-013. C57BL/6 mice were subcutaneously injected with MC38 cells at the density of 2 × 10^5^. When MC38 tumors reached a mean size about 70 mm^3^, tumor-bearing mice were randomized into four groups (n = 7), with the difference of mean tumor volume between groups less than 1 mm^3^. Each group accepted intragastric administration of saline, regorafenib (10 mg/kg/day), rosuvastatin (50 mg/kg/day) and combination treatment, respectively. Tumor volume and body weight of each mouse were monitored every 2 days. Calculation was carried out as follows: Tumor volume (TV)= (length × width^2^)/2; Relative Tumor volume (RTV) = TV _day n_/TV _day 1_ × 100%; Inhibition ratio (IR) of Tumor: IR _Tumor_ = 100% - Tumor weight _treatment group_/Tumor weight _control group_ × 100%; T/C (Relative tumor proliferation rate) = RTV _treatment group_/RTV _control group_.

### 2.6 Intratumor protein extraction

About 0.01 g tumor tissue was weighed into centrifuge tubes with a small steel ball per tube, and 100 μL 1% NP40 lysate with phosphatase and proteasome inhibitors were added. Grind in a tissue grinder (60 Hz, 6 min), and then centrifuge at 15,000 rpm for 30 min at 4°C. Supernatant was removed into a new centrifugal tube and protein concentration was measured. Remove 200 μg protein from the supernatant, add 100 μL 5× loading buffer, and fix the volume to 200 μL with ddH2O. Samples were heated at 95°C for 30 min, and subsequently immunoblotting analysis was performed.

### 2.7 Statistical analysis

All experiments were repeated at least three times in our study, Data was analyzed by GraphPad Prism 8.0 and presented as mean ± SD. Two-tail Student’s *t*-test were used to analyze comparisons between groups and *p* < 0.05 were considered statistically significant (*: *p* < 0.05, **: *p* < 0.01, and ***: *p* < 0.001; n. s: no sense).

## 3 Results

### 3.1 Co-treatment with regorafenib/rosuvastatin synergistically suppressed cell proliferation of colorectal cancer cells *in vitro*

**FIGURE 1:**
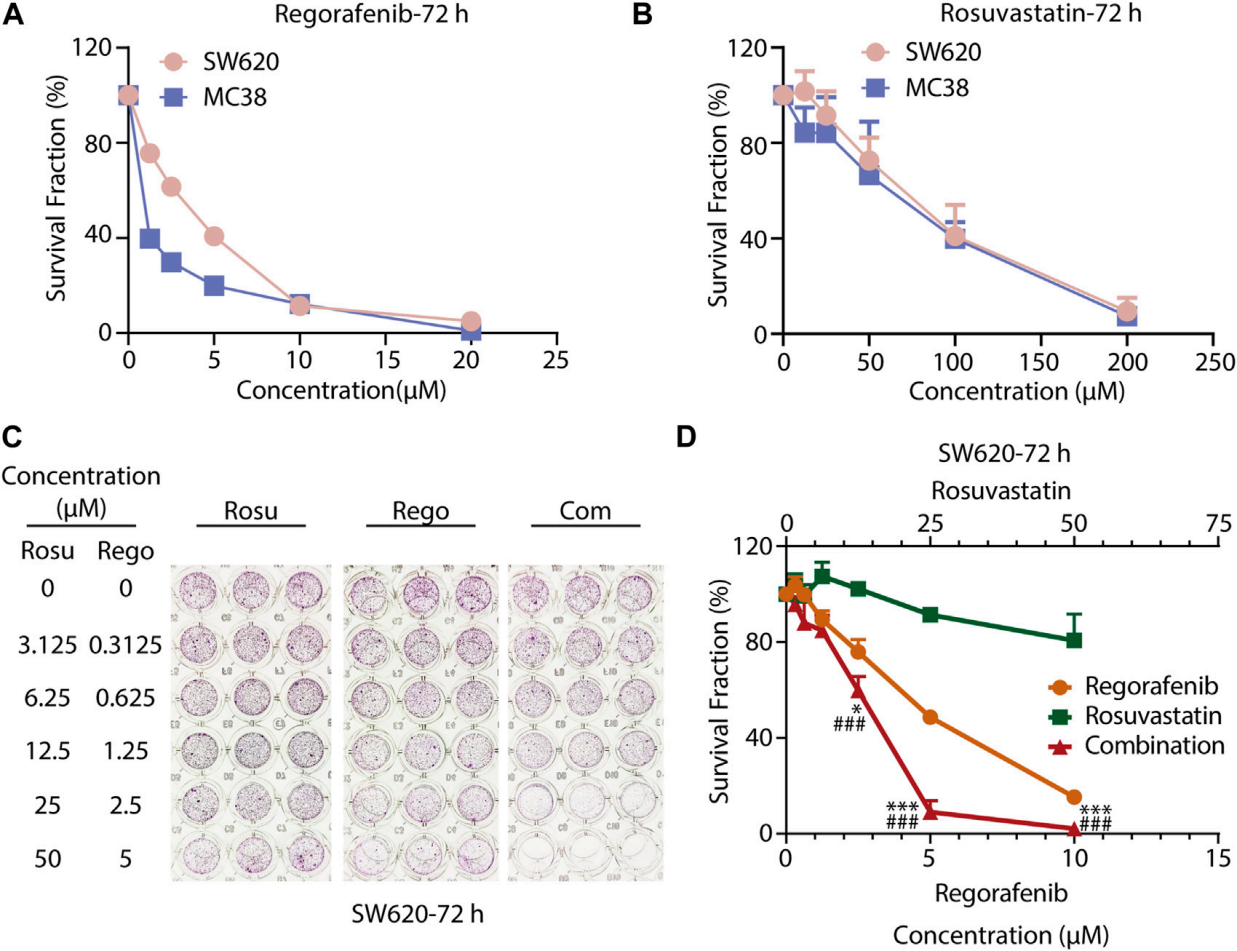
Co-treatment with regorafenib/rosuvastatin synergistically suppressed cell proliferation of colorectal cancer cells *in vitro*. **(A, B)** SRB assays to examine the effects of regorafenib (range, 1.25–20 μM) and rosuvastatin (range, 12.5–200 μM) on SW620 and MC38 cells proliferation for 72 h. **(C, D)** SRB assays to examine the effects of regorafenib, rosuvastatin, and co-treatment on SW620 cells proliferation for 72 h. The data were shown as mean ± SD of three replicate assays. *, *p* < 0.05; ***/###, *p* < 0.001 (*: *vs.* Regorafenib group; #: *vs.* Rosuvastatin group). Rosu: Rosuvastatin; Rego: Regorafenib; Com: Combination.

To evaluate the growth-inhibitory effects of regorafenib or rosuvastatin treatment on colorectal cancer cells, we treated SW620 and MC38 cells with serial concentrations of regorafenib (range, 1.25–20 μM) or rosuvastatin (range, 12.5–200 μM) for 72 h, then the cell viabilities were assessed by SRB assay. As shown in Figures 1A, B, both SW620 and MC38 showed a concentration-dependent sensitivity to regorafenib with the half maximal inhibitory concentration (IC_50_) values of 3.20 ± 0.46 μM and 1.07 ± 0.16 μM respectively, while rosuvastatin posed little effects on SW620 and MC38 cells growth, with IC_50_ values of 83.15 ± 20.75 μM and 72.11 ± 21.90 μM respectively (Supplementary Table S1).

To further investigate whether regorafenib and rosuvastatin posed synergistic antitumor effects in colorectal cancer cells, CRC cells were exposed with increasing concentrations of regorafenib and rosuvastatin for indicated times. As shown in Figures 1C, D; Supplementary Figure S1A, B; Supplementary Tables S2–S4, we found that regorafenib and rosuvastatin combination synergistically suppressed SW620 cells growth at 24, 48 and 72 h. To some extent, regorafenib in combination with rosuvastatin might reduce the concentrations of the two drugs, but retain the efficacy. For SW620 cells, when the anti-tumor activity reached 50% at 72 h, the concentrations of regorafenib and regorafenib in single treatment group were ∼5 μM and >50 μM respectively, while the concentrations of regorafenib and regorafenib in combination treatment group were ∼3 μM and ∼12.5 μM. Moreover, the synergistic anti-proliferative effects at 72 h were stronger than that at 24 h and 48 h, which was reflected by the gradually decreasing CDI values over time, suggesting a strengthened synergy over a time course. These data demonstrated the synergistic anti-proliferative effects between regorafenib and rosuvastatin in a time-dependent manner. Similar synergistic anti-proliferative effects were also observed on MC38 cells (Supplementary Figure S1C, D; Supplementary Table S5 **)**. Taken together, co-treatment with rosuvastatin and regorafenib induced a more pronounced growth inhibition in colorectal cancer cells compared to single-agent treatments *in vitro*, supporting the synergistic antitumor potential of regorafenib/rosuvastatin combination.

### 3.2 Combination regimen of regorafenib/rosuvastatin synergistically induced cell apoptosis of colorectal cancer cells *in vitro*

**FIGURE 2:**
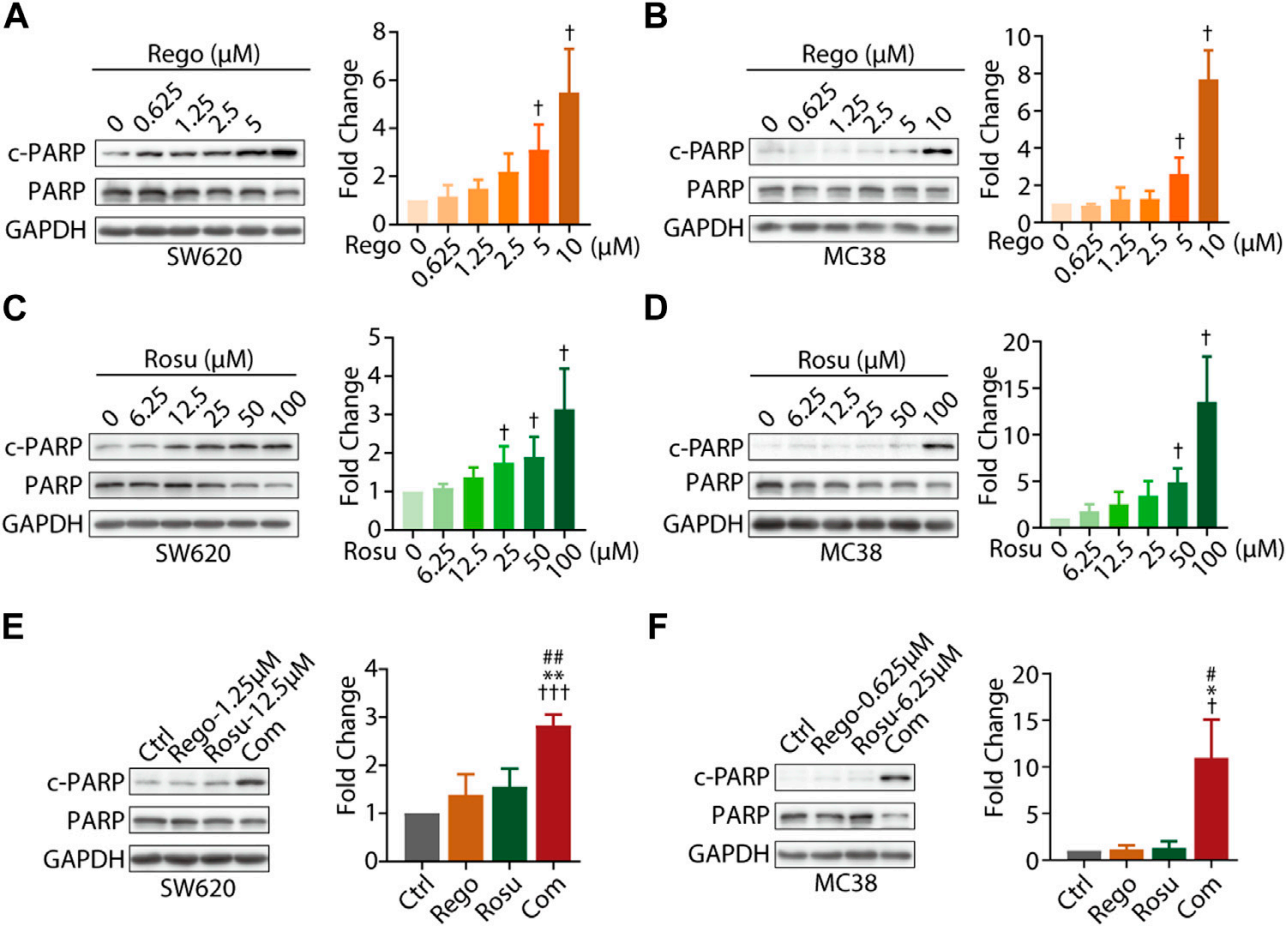
Combination regimen of regorafenib/rosuvastatin synergistically induced cell apoptosis of colorectal cancer cells *in vitro*. **(A**–**D)** Immunoblotting analysis cellular c-PARP/PARP expression levels of SW620 and MC38 cells treated with different concentration of regorafenib (range, 0.625–10 μM) or rosuvastatin (range, 6.25–100 μM) for 24 h. **(E, F)** Immunoblotting analysis on cellular c-PARP/PARP expression levels of SW620 and MC38 cells treated regorafenib (1.25 μM and 0.625 μM, respectively), rosuvastatin (12.5 μM and 6.25 μM, respectively) or combinational treatment for 24 h. The density measurement of immunoblotting analysis was performed by ImageJ. Indicated protein levels were determined relative to GAPDH protein levels and normalized relative to control cells. The data are shown as mean ± SD of three replicates assays. †/*/#, *p* < 0.05; **/##, *p* < 0.01; †††, *p* < 0.001 (†. *vs.* Control group; *: *vs.* Regorafenib group; #: *vs.* Rosuvastatin group). Rosu: Rosuvastatin; Rego: Regorafenib; Com: Combination.

As regorafenib was found to induce apoptosis of CRC cells, we next asked whether regorafenib/rosuvastatin co-treatment made difference in cellular apoptosis beyond proliferative inhibition (Chen et al., 2014). The classical apoptosis markers, cleavage of PARP (c-PARP), cleaved caspase 3 (c- Caspase 3), together with their full-length form, as well as the classical anti-apoptotic protein Bcl-2 were assessed in our study. First, we investigated the pro-apoptosis effects of regorafenib/rosuvastatin on SW620 and MC38 cells. As shown in Figures 2A, B, regorafenib induced a substantially concentration-dependent increase of c-PARP/PARP in SW620 and MC38 cells, along with the accumulation of c-Caspase 3/Caspase 3 and the decrease of Bcl-2 (Supplementary Figure S2A, B). At the same time, the similar pro-apoptosis effects were also observed in rosuvastatin-treated SW620 and MC38 cells (Figures 2C, B; Supplementary Figure S2C), indicating regorafenib and rosuvastatin induced apoptosis in CRC cells.

To further examine whether co-treatment with regorafenib/rosuvastatin synergistically induced CRC apoptosis, we also investigated apoptosis related proteins in regorafenib/rosuvastatin co-treatment group. As depicted in Figures 2E, F, low-concentration regorafenib/rosuvastatin co-treatment group displayed a significant boost of c-PARP/PARP expression levels in SW620 and MC38 cells, whereas almost no pro-apoptotic effects were observed in low-concentration regorafenib or rosuvastatin mono-treatment. Moreover, we also introduced apoptotic inhibitor Z-VAD-FMK and found Z-VAD-FMK reversed the pro-apoptotic effects induced by regorafenib and rosuvastatin combination, these data collectively demonstrated that regorafenib in combination with rosuvastatin synergistically induced the apoptosis of colorectal cancer *in vitro* (Supplementary Figure S2D).

### 3.3 Regorafenib/rosuvastatin co-treatment exerted synergistical antitumor effect by inhibiting intrinsic MAPK signaling pathway

Furthermore, we attempted to explore the molecular mechanisms underlying the synergistic effects of regorafenib and rosuvastatin. As an active multi-kinase inhibitor, regorafenib is capable of potently suppressing mitogen-activated protein kinase (MAPK) signaling pathway, thus inhibiting tumor progression. Therefore, we speculated regorafenib/rosuvastatin co-treatment might exert a synergistic antitumor effect by amplifying inhibitory effect on MAPK signaling.

**FIGURE 3:**
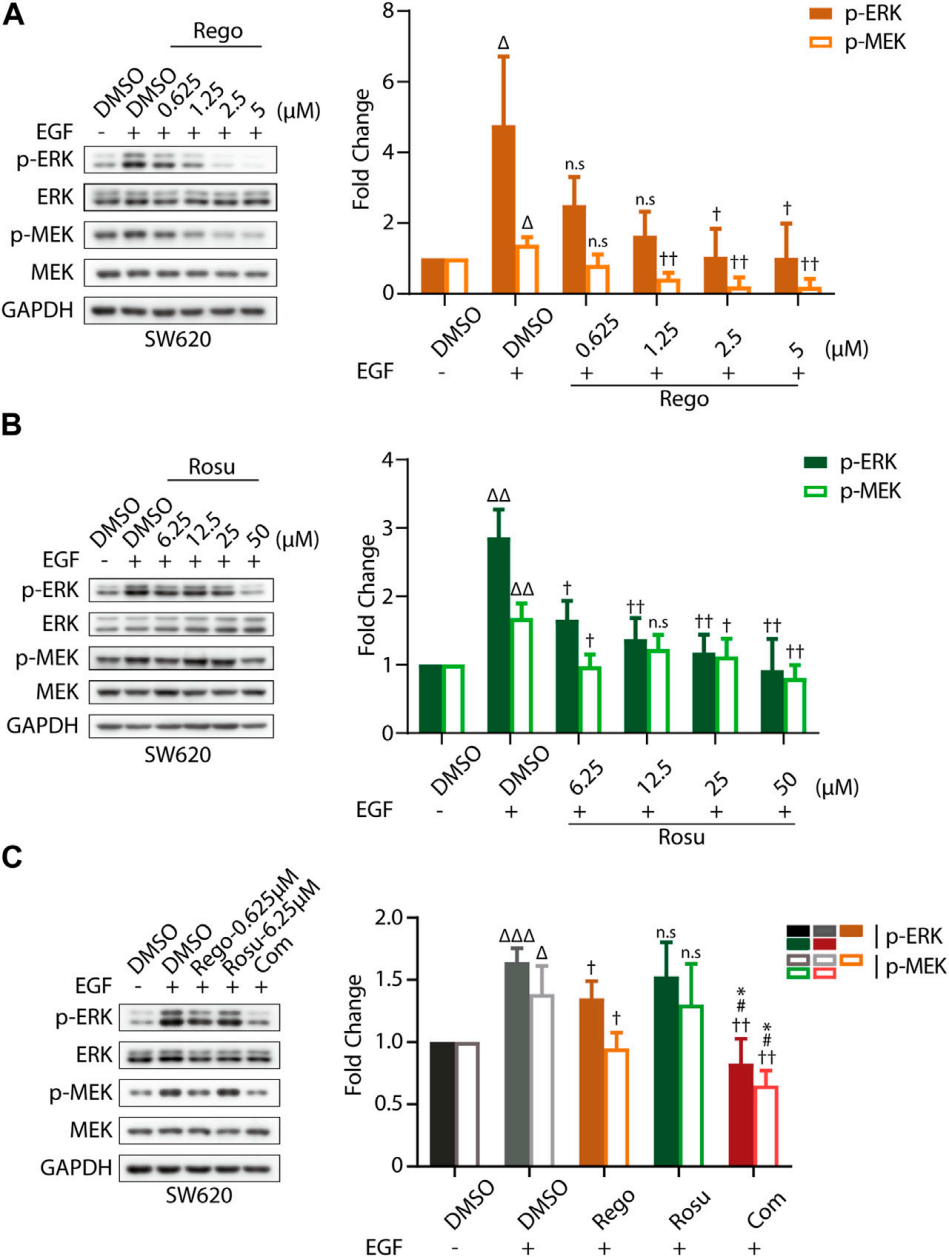
Regorafenib/rosuvastatin co-treatment exerted synergistical antitumor effect by inhibiting intrinsic MAPK signaling pathway. **(A, B)** Immunoblotting analysis to examine p-ERK/ERK and p-MEK/MEK expression levels in SW620 cells treated with regorafenib (range, 0.625–5 μM) or rosuvastatin (range, 6.25–50 μM). **(C)** Immunoblotting analysis to examine p-ERK/ERK and p-MEK/MEK expression levels in SW620 cells treated with regorafenib (0.625 μM), rosuvastatin (6.25 μM) and combinational treatment. SW620 cells were starved in serum-free RPMI-1640 media overnight and then treated with regorafenib/rosuvastatin or DMSO for 4 h. Before harvested, cells were treated with 500 ng/mL EGF for 30 min. The data are shown as mean ± SD of three replicates assays. n. s, no sense; †/Δ/*/#, †/*p* < 0.05; ††/ΔΔ, *p* < 0.01; ΔΔΔ, *p* < 0.001 (†: *vs*. EGF + DMSO group; Δ: *vs*. DMSO group; *: *vs.* Regorafenib group; #: *vs.* Rosuvastatin group). Rosu: Rosuvastatin; Rego: Regorafenib; Com: Combination.

First, we evaluated cellular MAPK pathway in response to regorafenib and/or rosuvastatin treatment in SW620 cells. As shown in Supplementary Figures S3A, B, regorafenib induced a remarkable gradient-dependent inhibition of the levels of p-ERK/ERK and p-MEK/MEK but rosuvastatin only induced a slight inhibition at higher concentrations. Then, we further examined the synergistic effects and found that regorafenib in combination with rosuvastatin effectively inhibited p-ERK/ERK and p-MEK/MEK levels (Supplementary Figure S3C). To further demonstrate this conclusion, epidermal growth factor (EGF), a classical stimulator of MAPK signaling, was introduced in our study and we found that EGF stimulation significantly upregulated MAPK signaling (Figures 3A–C), which was consistent with previous studies (Stefani et al., 2021). Subsequently, we further examined the inhibitory effects of regorafenib and rosuvastatin on MAPK signaling triggered by EGF. As shown in Figures 3A,B, regorafenib still posed a significantly gradient-dependent inhibition of the levels of p-MEK/p-ERK and rosuvastatin posed an obvious inhibitory effect at high concentrations. Intriguingly, regorafenib and rosuvastatin co-treatment displayed a more significant downregulation of the levels of p-MEK/p-ERK (Figure 3C). These results collectively demonstrated that regorafenib and rosuvastatin synergistically posed antitumor activity by inhibiting intrinsic MAPK signaling pathway.

### 3.4 Rosuvastatin significantly strengthened the antitumor effects of regorafenib on colorectal cancer *in vivo*

Aforementioned data has demonstrated that regorafenib in combination with rosuvastatin synergistically inhibited colorectal cancer growth *in vitro*, so we next attempted to investigate whether regorafenib and rosuvastatin co-treatment significantly inhibited colorectal cancer progression *in vivo*. To determine the doses of regorafenib and rosuvastatin in animal studies, we referred to previous reports and chose the suitable doses with regorafenib as 10 mg/kg, and rosuvastatin as 50 mg/kg. MC38 cells were subcutaneously injected into C57BL/6 mice, after tumor volume reached ∼70 mm^3^, these mice were divided into four groups randomly (n = 7/group), and daily intragastrical administered with regorafenib (10 mg/kg/day) and/or rosuvastatin (50 mg/kg/day), the tumor volume and body weight were evaluated and recorded every 2 days.

**FIGURE 4:**
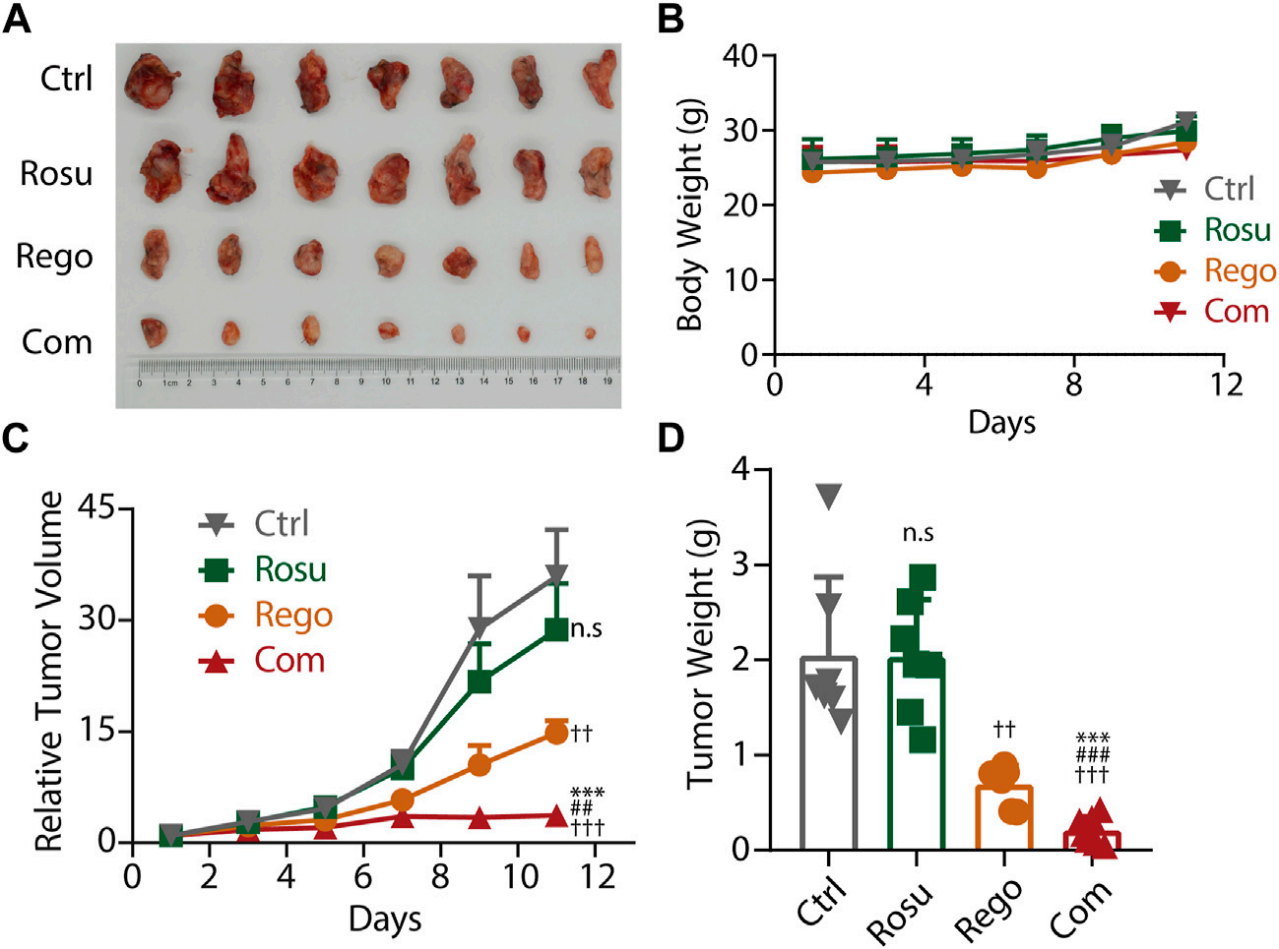
Rosuvastatin significantly promoted the antitumor effects of regorafenib on colorectal cancer *in vivo*. **(A)** Representative tumor photograph of each group. **(B)** Body weight of mice in each group. **(C)** Relative tumor volume of each group. **(D)** Tumor weight of each group at the endpoint of experiment. C57BL/6 mice accepted subcutaneous injection of MC38 cells. When MC38 tumors grew up to ∼70 mm^3^, tumor-bearing mice were randomized into four groups (n = 7/group), guaranteeing the difference in mean tumor volume between groups less than 1 mm^3^. Each group mice accepted intragastric administration of saline, regorafenib (10 mg/kg/day), rosuvastatin (50 mg/kg/day) and combination treatment, respectively. Tumor volume and body weight were recorded every 2 days. The quantitative data are presented as mean ± SEM. n. s, no sense; ††/##, *p* < 0.01; †††/###/***, *p* < 0.001 (†: *vs*. saline group, *: *vs*. Regorafenib group; #: *vs*. Rosuvastatin group). Rosu: Rosuvastatin; Rego: regorafenib; Com: Combination.

As shown in Figures 4A, B, regorafenib and rosuvastatin co-treatment significantly inhibited the growth of MC38 tumors and the inhibition ratio (IR) was 89.79%, while the inhibition rate of regorafenib group and rosuvastatin group was 66.23% and 0.60%, respectively. Meanwhile, a significant reduction in relative tumor volume (RTV) was also observed in regorafenib and rosuvastatin co-treatment group and the T/C value was 10.44%, while the T/C values of regorafenib group and rosuvastatin group were 41.26% and 79.87% (quantification in Supplementary Table S6), respectively. These data collectively demonstrated regorafenib and rosuvastatin co-treatment significantly inhibited MC38 tumors’ growth *in vivo*. To reflect the potential impact on animal survival from the side, we evaluated the tumor growth delay effect by calculating the average tumor growth days to reach approximately 4 times in the initial tumor volume for each group. As shown in Supplementary Figure S4. The tumors bearing on control mice took 5 days to reach 4 times, rosuvastatin mice took ∼5 days, regorafenib mice took less than 7 days, while regorafenib and rosuvastatin combination mice took more than 11 days to reach 4 times in initial volume, suggesting regorafenib in combination with rosuvastatin was likely to improve animal survival.

### 3.5 Regorafenib and rosuvastatin co-treatment synergistically suppressed MAPK signaling and promoted apoptosis of colorectal cancer *in vivo*

**FIGURE 5:**
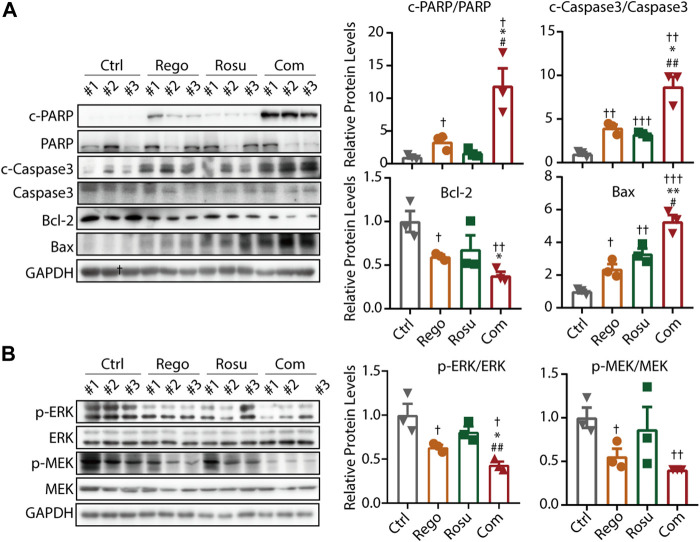
Regorafenib and rosuvastatin co-treatment synergistically suppressed MAPK signaling and promoted apoptosis of colorectal cancer *in vivo*. **(A)** Immunoblotting analysis to examine intratumor c-PARP/PARP expression levels of MC38 tumors. **(B)** Immunoblotting analysis to examine intratumor p-ERK/ERK expression levels of MC38 tumors. The data are shown as mean ± SD, n. s, no sense, †/*/#, *p* < 0.05; ††/**/##, *p* < 0.01; †††, *p* < 0.001 (†: *vs*. Control group; *: *vs*. Regorafenib group; #: *vs*. Rosuvastatin group). Rosu: Rosuvastatin; Rego: Regorafenib; Com: Combination.

To validate the finding that regorafenib and rosuvastatin combination therapy synergistically suppressed MAPK signaling and induced the apoptosis of colorectal cancer *in vitro*, we next examined intratumor MAPK signaling and apoptosis related protein levels in tumor samples. As shown in Figure 5A, regorafenib in combination with rosuvastatin synergistically promoted the expression of c-PARP/PARP, c-Caspase3/Caspase3 and Bax, but significantly downregulated Bcl-2 levels, these data collectively demonstrated that regorafenib in combination with rosuvastatin synergistically induced the apoptosis of colorectal cancer *in vivo*. Meanwhile, we also investigated intratumor MAPK signaling related protein levels and found regorafenib and rosuvastatin co-treatment synergistically suppressed intratumor MAPK signaling, which was consistent with results of cellular experiments *in vitro* (Figure 5B).

Collectively, these findings corroborated the notion that regorafenib and rosuvastatin co-treatment synergistically suppressed MAPK signaling and induces apoptotic cell death, thus impeding colorectal cancer growth *in vitro* and *in vivo*.

## 4 Discussion

Despite the great progress in target therapies in clinical treatment of advanced colorectal cancer patients, the multi-kinase inhibitors including regorafenib are yet challenged by the limited efficacy (Argiles et al., 2015; Lau et al., 2021). Therefore, it is urgent to develop potent combined therapies to enhance the antitumor activity of regorafenib for clinical treatment of colorectal cancer. In our study, we demonstrated that regorafenib in combination with rosuvastatin significantly synergistic inhibited of colorectal cancer growth *in vitro* and *in vivo*.

Regorafenib functions as a small-molecular multi-targets inhibitor of various membrane-bound and intracellular kinases involved in normal/pathologic cellular progress, including tumorigenesis, angiogenesis, and maintenance of tumor microenvironment, *etc.* (Wilhelm et al., 2011) Regorafenib is mainly transformed into active metabolites M-2 and M-5, thus inhibiting multiple kinases, including VEGFR 1–3, KIT, PDGFR-alpha/beta, FGFR1/2, RET, *etc.* (Ettrich and Seufferlein, 2018) Up to now, regorafenib has been widely utilized to treatment colorectal cancer, especially metastatic colorectal cancer (Van Cutsem et al., 2019; Sun et al., 2021). An international clinical trial was performed to test the efficacy of regorafenib in patients with metastatic colorectal cancer progressing after all approved standard therapies and found the median overall survival was 6.4 months in the regorafenib group while that of placebo group was 5.0 months (Grothey et al., 2013). In addition, regorafenib in combination with FOLFIRI as second-line treatment of metastatic colorectal cancer was also assessed in a phase II trial. The clinical trial results showed that median progression-free survival (PFS) was 6.14 months for FOLFIRI and regorafenib co-treatment group *versus* 5.29 months for FOLFIRI alone group, and median overall survival (OS) were 13.2 months and 12 months respectively (Ettrich and Seufferlein, 2018), suggesting regorafenib in combination with FOLFIRI could slightly improve the PFS and OS of metastatic colorectal cancer patients compared to FOLFIRI alone. These clinical trials collectively suggested the potential possibility of regorafenib for treatment metastatic colorectal cancer patients, but the efficacy remains to be improved, by newly-developed combination regimens.

Statins function as lipid-lowering agents in clinical by competitively inhibiting 3-hydroxy-3-methylglutaryl-CoA reductase (HMGCR) mediating production of mevalonate therefore instrumentally interfering with endogenous cholesterol synthesis (Smit et al., 2020). Mounting evidence obtained from animal studies or observation on patients taking statins revealed that statins could lower the risk of cancer and improve the prognosis of cancer patients, with the mechanisms remained elusive (Iannelli et al., 2018; Jiang et al., 2021). Mevalonate, the production of HMGCR, is further metabolized into geranylgeranyl pyrophosphate (GGPP) and farnesyl pyrophosphate (FPP), which play fundamental roles in posttranslational modifications (PTMs) of proteins, especially prenylation (Gobel et al., 2020). Prenylation promotes KRAS, one of the most frequently mutated genes in colorectal cancer, transforming from GDP-bound inactive state into GTP-bound active state anchored in plasma membrane, then initiating downstream signaling (Liu et al., 2019b; Wang et al., 2021). Hence, inhibition of the mevalonate pathway disrupts the prenylation of KRAS, and therefore inhibits KRAS activity (Nam et al., 2021). In addition, inhibition of cholesterol synthesis also directly affects the membrane localization of lipophilic proteins like RAS by impeding the dynamic assemblies of sphingolipids, cholesterol, and other components into “lipid rafts” (Ferreira et al., 2022; Yeagle, 2022), which suggests the possibility that rosuvastatin prevents KRAS protein from membrane anchoring and suppresses its downstream signaling pathway activation. In addition, it has been reported that statins can uphold reactive oxygen species (ROS) production by curbing the mevalonate pathway, consequently suppressing activation of Akt and ERK pathways (Qi et al., 2013). Taken together, we speculated regorafenib in combination with rosuvastatin might pose synergistic antitumor activity through MAPK signaling. In our study, we indeed demonstrated rosuvastatin synergized with regorafenib in blocking MEK-ERK signaling, therefore synergistic inhibiting colorectal cancer growth *in vitro* and *in vivo*.

Multiple studies have revealed that MAPK signaling pathway is widely involved in the process of cellular apoptosis and the activation of MAPK signaling inhibits apoptosis through complicated mechanisms. On the one hand, it suppresses of the function of pro-apoptotic proteins. For example, MEK1/2 or ERK1/2 activation inhibits Bim activity by phosphorylating Bim at Ser109/Thr110 and downregulates its expression at the same time (Biswas and Greene, 2002). Moreover, MAPK activation also mediates the phosphorylation and inactivation of Bcl-2 family member Bad at Ser112 in an p90 ribosomal S6 kinase (RSK) dependent manner (Sturgill et al., 1988). On the other hand, MAPK signaling also promotes the activity of anti-apoptotic molecules. ERK1/2 phosphorylates MCL1 at Thr163 and increases its stability and anti-apoptotic activity (Domina et al., 2004). These studies collectively indicate that MAPK signaling is closely associated with apoptosis. Based on the results that regorafenib and rosuvastatin combination synergistically suppressed MAPK signaling, we further examined the effects on apoptosis and found regorafenib in combination with rosuvastatin synergistically induced the apoptosis of colorectal cancer *in vitro* and *in vivo*.

According to pharmacokinetics studies, most statins are the substrates of CYP3A4, P-glycoprotein and/or OATP1B1 transporters, whose combined utilization with the inducers or inhibitors of these transporters would result in changing plasma concentrations and inducing toxicity, but rosuvastatin is not included (Balasubramanian and Maideen, 2021). So, although researches shows that regorafenib is mainly metabolized by CYP3A4 (Kubota et al., 2020), suggesting regorafenib may affect the metabolism of some statins metabolizing by CYP3A4 like simvastatin and atorvastatin, the combination of regorafenib and rosuvastatin may pose little effects on their metabolism *in vivo*. The toxicity of statins includes elevated liver enzymes, acute kidney injury, hepatotoxicity, etc (Famularo et al., 2007; Balasubramanian and Maideen, 2021). Some studies have revealed that regorafenib also caused hepatotoxicity (Sacre et al., 2016), suggesting the potential overlapping safety concerns regarding this combination of drugs, which needs special attention if the combination regimen is applied to clinical colorectal cancer treatment. In addition, multiple studies have demonstrated that the interaction of rosuvastatin with darunavir/ritonavir, erythromycin, fluconazole, itraconazole and antacid was considered statistically and clinically relevant (Kanukula et al., 2021), but the pharmacokinetic drug-drug interactions between regorafenib and rosuvastatin have not been reported, which needs to be further investigated in the future.

Our findings demonstrated the synergistic antitumor activity of regorafenib and rosuvastatin combination therapy in clinical colorectal cancer *in vitro* and *in vivo*. Mechanically, regorafenib in combination with rosuvastatin synergistically suppressed MAPK signaling, thus suppressing cell proliferation. In addition, regorafenib and rosuvastatin combination therapy synergistically induced the apoptosis of colorectal cancer *in vitro* and *in vivo*. Our study clarified that non-clinical studies demonstrated synergy between regorafenib and rosuvastatin. Therefore, it may potentially be evaluated as a novel combination regimen for clinical treatment of colorectal cancer.

## Data Availability

The original contributions presented in the study are included in the article/Supplementary Material, further inquiries can be directed to the corresponding author.
