# Supplementary material for: Synergistic antitumor activity of regorafenib and rosuvastatin in colorectal cancer
Source: Front Pharmacol. 2023 Apr 17;14:1136114. doi: 10.3389/fphar.2023.1136114 (PMC10149949; doi:10.3389/fphar.2023.1136114)
Supplement: Supplementary file 1 [file DataSheet1.docx]

Supplementary Material

Synergistic Antitumor Activity of Regorafenib and Rosuvastatin in Colorectal Cancer

Tao Yuan^1, #^, Ruilin Wu^1, #^, Weihua Wang^1^, Yue Liu^1^, Wencheng Kong^2^, Bo Yang^1^, Qiaojun He^1, 3, 5^, Hong Zhu^1,4, 5^

*** Correspondence:** Dr. Hong Zhu, Zhejiang Province Key Laboratory of Anti-Cancer Drug Research, College of Pharmaceutical Sciences, Zhejiang University, 866# Yuhangtang Rd, Hangzhou, Zhejiang 310058, China, email: hongzhu@zju.edu.cn; fax/tel: 86571-88208400

# Supplementary Tables

**Table S1. IC_50_ values of Regorafenib and Rosuvastatin on CRCs**

| IC_50_ | Regorafenib (μM） | Rosuvastatin (μM） |
| --- | --- | --- |
| SW620 | 3.20 ± 0.46 | 83.15 ± 20.75 |
| MC38 | 1.07 ± 0.16 | 72.11 ± 21.90 |

**Table S2. CDI of regorafenib in combination with rosuvastatin on SW620 for 24 h**

| **Regorafenib** | | **Rosuvastatin** | | **Combination** | | |
| --- | --- | --- | --- | --- | --- | --- |
| **Con (μM)** | **IR (%)** | **Con (μM)** | **IR (%)** | **Combination Ratio** | **IR（%）** | **CDI** |
|  |  |  |  | **(Rego: Rosu)** |  |  |
| 10 | 33.27 | 50 | 13.28 | 1:5 | 50.65 | 0.85 |
| 5 | 21.60 | 25 | 7.60 |  | 23.83 | 1.05 |
| 2.5 | 15.84 | 12.5 | 9.64 |  | 12.32 | 1.15 |
| 0 | 0.00 | 0 | 0.00 |  | 0.00 | - |

CDI: coefficient of drug interaction; Con: concentration; IR: inhibit ratio of SRB; Rego: Regorafenib; Rosu: Rosuvastatin;

**Table S3. CDI of regorafenib in combination with rosuvastatin on SW620 for 48 h**

| **Regorafenib** | | **Rosuvastatin** | | **Combination** | | |
| --- | --- | --- | --- | --- | --- | --- |
| **Con (μM)** | **IR (%)** | **Con (μM)** | **IR (%)** | **Combination Ratio** | **IR（%）** | **CDI** |
|  |  |  |  | **(Rego: Rosu)** |  |  |
| 10 | 58.42 | 50 | 10.40 | 1:5 | 84.21 | 0.42 |
| 5 | 31.59 | 25 | 6.18 |  | 36.59 | 0.99 |
| 2.5 | 21.88 | 12.5 | 3.33 |  | 21.00 | 1.05 |
| 0 | 0.00 | 0 | 0.00 |  | 0.00 | - |

CDI: coefficient of drug interaction; Con: concentration; IR: inhibit ratio of SRB; Rego: Regorafenib; Rosu: Rosuvastatin;

**Table S4. CDI of regorafenib in combination with rosuvastatin on SW620 for 72 h**

| **Regorafenib** | | **Rosuvastatin** | | **Combination** | | |
| --- | --- | --- | --- | --- | --- | --- |
| **Con (μM)** | **IR (%)** | **Con (μM)** | **IR (%)** | **Combination Ratio**  **(Rego: Rosu)** | **IR（%）** | **CDI** |
| 10.00 | 84.74 | 50.00 | 19.35 | 1: 5 | 97.91 | 0.17 |
| 5.00 | 51.40 | 25.00 | 8.74 |  | 90.95 | 0.20 |
| 2.50 | 24.20 | 12.50 | (2.11) |  | 40.24 | 0.77 |
| 1.25 | 10.68 | 6.25 | (7.37) |  | 15.19 | 0.88 |
| 0.63 | 0.30 | 3.13 | 0.73 |  | 12.16 | 0.89 |
| 0.31 | (3.60) | 1.56 | (5.77) |  | 4.32 | 0.87 |
| 0.00 | 0.00 | 0.00 | 0.00 |  | 0.00 | - |

( ) : represented negative numbers; CDI: coefficient of drug interaction; Con: concentration; IR: inhibit ratio of SRB; Rego: Regorafenib; Rosu: Rosuvastatin;

**Table S5. CDI of regorafenib in combination with rosuvastatin on MC38 for 24 h**

| **Regorafenib** | | **Rosuvastatin** | | **Combination** | | |
| --- | --- | --- | --- | --- | --- | --- |
| **Con (μM)** | **IR (%)** | **Con (μM)** | **IR (%)** | **Combination Ratio** | **IR（%）** | **CDI** |
|  |  |  |  | **(Rego: Rosu)** |  |  |
| 5 | 41.74 | 200 | 23.16 | 1:40 | 85.64 | 0.32 |
| 2.5 | 25.62 | 100 | 9.14 |  | 46.05 | 0.80 |
| 1.25 | 17.71 | 50 | 6.30 |  | 21.61 | 1.02 |
| 0 | 0.00 | 0 | 0.00 |  | 0.00 | - |

CDI: coefficient of drug interaction; Con: concentration; IR: inhibit ratio of SRB; Rego: Regorafenib; Rosu: Rosuvastatin;

**Table S6. Inhibitory effect of regorafenib and/or rosuvastatin in MC38 tumors**

| Groups | Tumor Weight (g) | IR (%) | RTV | T/C (%) |
| --- | --- | --- | --- | --- |
| Control | 2.04 ± 0.31 | - | 35.92 ± 6.30 | - |
| Rosuvastatin | 2.03 ± 0.23 | 0.60 | 28.69 ± 6.26 | 79.87 |
| Regorafenib | 0.69 ±0.08 | 66.23 | 14.82 ± 1.64 | 41.26 |
| Combination | 0.21 ± 0.05 | 89.79 | 3.75 ± 0.29 | 10.44 |

IR: inhibit ratio of tumor; T/C: relative tumor proliferation rate.

# Supplementary Figures


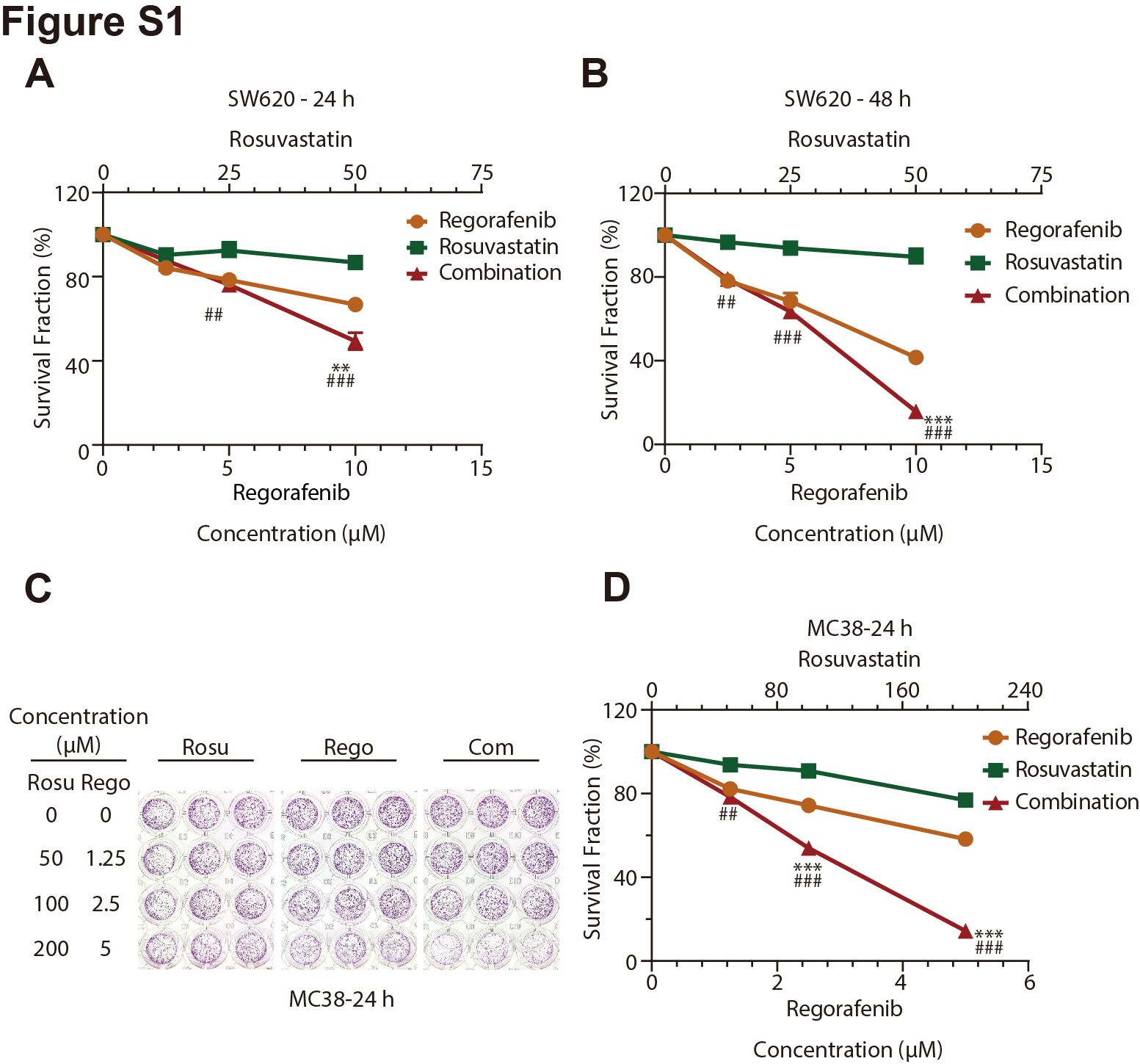


**Figure S1.** **Regorafenib/rosuvastatin co-treatment synergistically suppressed cell proliferation of colorectal cancer cells *in vitro*.**

**(A-B)** SRB assays to examine the effects of regorafenib, rosuvastatin, and co-treatment on SW620 cells proliferation for 24 h and 48 h. (**C**-**D**) SRB assays to examine the effects of regorafenib, rosuvastatin, and co-treatment on MC38 cells proliferation for 24 h. The data were shown as mean ± SD of three replicate assays. **, *P* < 0.01; ***/###, *P* < 0.001 (*: *vs.* Regorafenib group; #: *vs.* Rosuvastatin group). Rosu: Rosuvastatin; Rego: Regorafenib; Com: Combination.


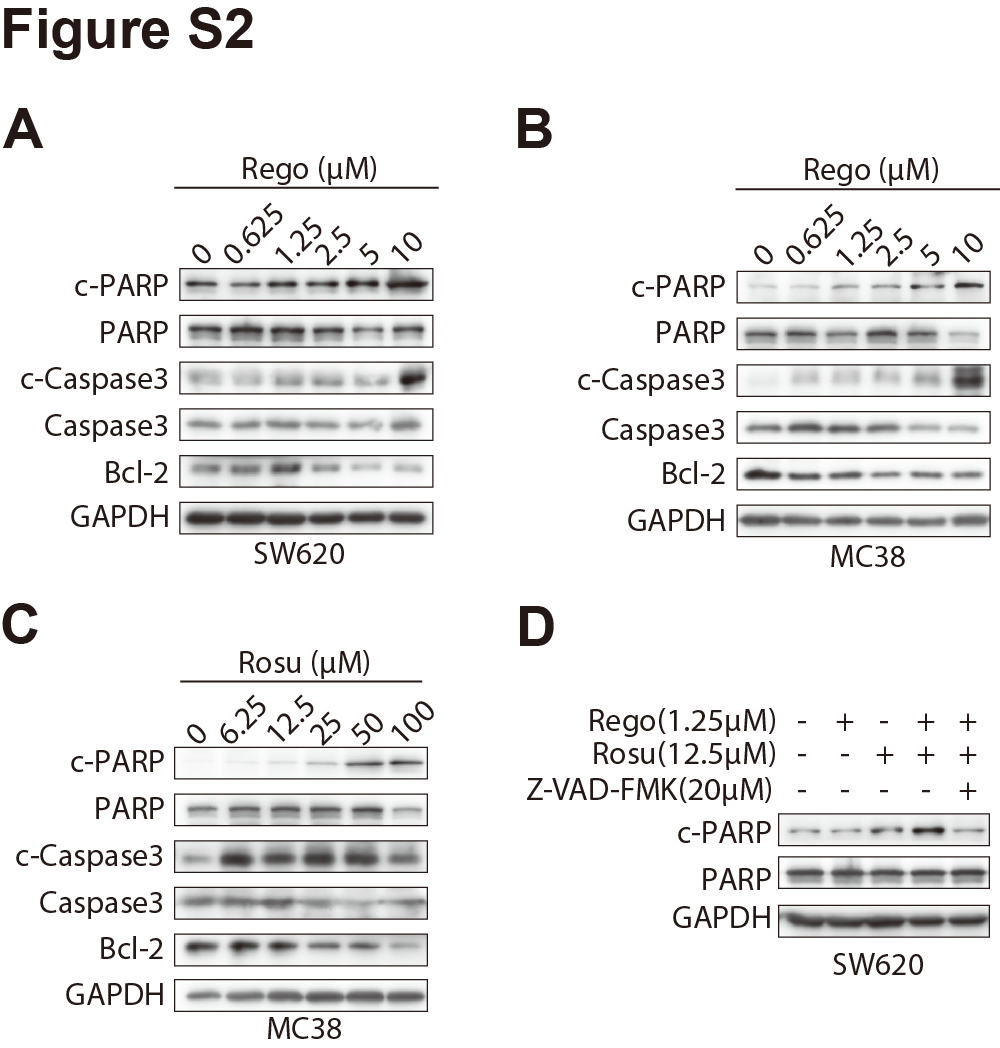


**Figure S2. Combination regimen of regorafenib/rosuvastatin synergistically induced cell apoptosis of colorectal cancer cells *in vitro*.**

(**A**-**C**) Immunoblotting analysis cellular c-PARP/PARP, c-Caspase 3/Caspase 3, Bcl-2 expression levels of SW620 and MC38 cells treated with different concentration of regorafenib (range, 0.625-10 μM) or rosuvastatin (range, 6.25-100 μM) for 24 h. (**D**) Immunoblotting analysis on cellular c-PARP/PARP, c-Caspase 3/Caspase 3, Bcl-2 expression levels of SW620 cells treated regorafenib (1.25 μM and 0.625 μM, respectively), rosuvastatin (12.5 μM and 6.25 μM, respectively) and combinational treatment plus apoptotic inhibition Z-VAD-FMK or not for 24 h. Rosu: Rosuvastatin; Rego: Regorafenib; Com: Combination.


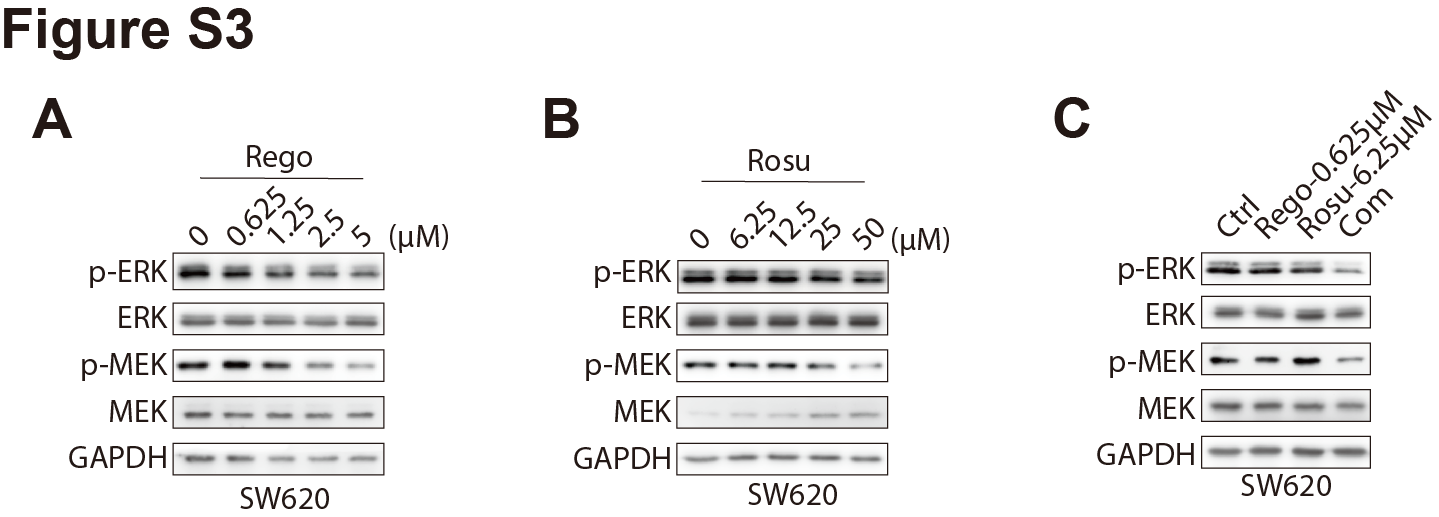


**Figure S3. Regorafenib/rosuvastatin co-treatment exerted synergistical antitumor effect by inhibiting intrinsic MAPK signaling pathway.**

(**A**-**B**) Immunoblotting analysis to examine p-ERK/ERK and p-MEK/MEK expression levels in SW620 cells treated with regorafenib (range, 0.625-5 μM) or rosuvastatin (range, 6.25-50 μM). (**C**) Immunoblotting analysis to examine p-ERK/ERK and p-MEK/MEK expression levels in SW620 cells treated with regorafenib (0.625 μM), rosuvastatin (6.25 μM) and combinational treatment. Rosu: Rosuvastatin; Rego: Regorafenib; Com: Combination.


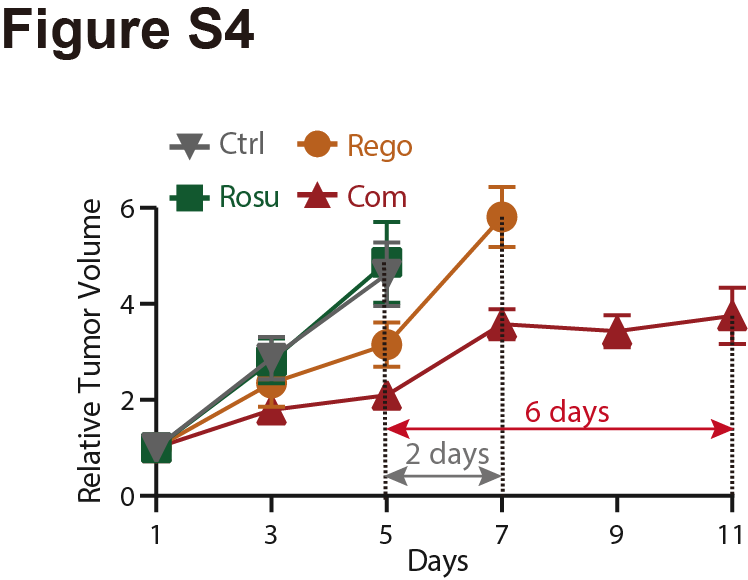


**Figure S4. Regorafenib/Rosuvastatin co-treatment was likely to improve animal survival.**
